# Dietary Vitamin D Increases Percentages and Function of Regulatory T Cells in the Skin-Draining Lymph Nodes and Suppresses Dermal Inflammation

**DOI:** 10.1155/2016/1426503

**Published:** 2016-09-08

**Authors:** Shelley Gorman, Sian Geldenhuys, Melinda Judge, Clare E. Weeden, Jason Waithman, Prue H. Hart

**Affiliations:** Telethon Kids Institute, University of Western Australia, Perth, WA, Australia

## Abstract

Skin inflammatory responses in individuals with allergic dermatitis may be suppressed by dietary vitamin D through induction and upregulation of the suppressive activity of regulatory T (T_Reg_) cells. Vitamin D may also promote T_Reg_ cell tropism to dermal sites. In the current study, we examined the capacity of dietary vitamin D_3_ to modulate skin inflammation and the numbers and activity of T_Reg_ cells in skin and other sites including lungs, spleen, and blood. In female BALB/c mice, dietary vitamin D_3_ suppressed the effector phase of a biphasic ear swelling response induced by dinitrofluorobenzene in comparison vitamin D_3_-deficient female BALB/c mice. Vitamin D_3_ increased the percentage of T_Reg_ (CD3+CD4+CD25+Foxp3+) cells in the skin-draining lymph nodes (SDLN). The suppressive activity of T_Reg_ cells in the SDLN, mesenteric lymph nodes, spleen, and blood was upregulated by vitamin D_3_. However, there was no difference in the expression of the naturally occurring T_Reg_ cell marker, neuropilin, nor the expression of CCR4 or CCR10 (skin-tropic chemokine receptors) on T_Reg_ cells in skin, SDLN, lungs, and airway-draining lymph nodes. These data suggest that dietary vitamin D_3_ increased the percentages and suppressive activity of T_Reg_ cells in the SDLN, which are poised to suppress dermal inflammation.

## 1. Introduction

Vitamin D plays an intrinsic role in shaping innate and adaptive immune responses [1, 2]. Vitamin D is produced following skin exposure to ultraviolet B photons of sunlight, resulting in the conversion of the precursor 7-dehydrocholesterol into vitamin D_3_, which can also be acquired through dietary supplementation. The vitamin D-binding protein (VDBP) transports much of this vitamin D_3_ into the liver, where a hydroxylation reaction converts vitamin D_3_ into 25-hydroxyvitamin D_3_ (25(OH)D_3_). This form of vitamin D_3_ is found at nanomolar levels in blood, and because of its relative stability and longer half-life, it is used as a measure of vitamin D sufficiency, with 50 nM currently considered the tipping point for insufficiency by the National Institute of Health [3] (although this remains controversial [4]). In renal proximal tubule epithelial cells, and other cells including disease-activated macrophages (reviewed in [5]), 25(OH)D_3_ is converted into the most active vitamin D metabolite, 1,25-dihydroxyvitamin D_3_ (1,25(OH)_2_D_3_). It is this form of vitamin D_3_ which has the most potent effects on regulating immune responses, with circulating levels in the picomolar range [1, 6].

Central to the ability of 1,25(OH)_2_D_3_ to modulate immune responses are changes to regulatory T cells (T_Reg_ cells) and dendritic cells (DCs) [7]. Topical (skin) application of 1,25(OH)_2_D_3_ enhanced the suppressive capacity [8, 9] and proliferative activity [10] of CD4+CD25+Foxp3+ T_Reg_ cells. Stimulation of DCs with bacterial products like lipopolysaccharide or cytokines like transforming growth factor-ß may result in the synthesis of 1,25(OH)_2_D_3_ from circulating 25(OH)D_3_, promoting T_Reg_ cell activity (reviewed in [1, 2]). The VDBP may also play an important role in this process, whereby high affinity VDBP can prevent conversion of 25(OH)D_3_ to 1,25(OH)_2_D_3_ by DCs and thus their ability to modulate T_Reg_ cell activity [11]. With the right costimulators, including interleukin-2, 1,25(OH)_2_D_3_ can modulate the suppressive functions of T_Reg_ cells independently of DCs [12].

While the capacity for 1,25(OH)_2_D_3_ to regulate adaptive immunity through its effects on T_Reg_ cells and DCs is clear, most studies have used supraphysiological levels of 1,25(OH)_2_D_3_ (≥10 nM). During monocyte differentiation into macrophages, increased concentrations of 1,25(OH)_2_D_3_ (up to 1 nM) were detected in cell culture media, but this was not observed during monocyte differentiation to DCs [13]. This increased production of 1,25(OH)_2_D_3_ could have paracrine effects on colocated DCs [13] and T cells [14]. However, most* in vitro* studies have used substantially more 1,25(OH)_2_D_3_ (≥10 nM) to modulate DC and T cell phenotype and function. T_Reg_ cell numbers and/or their suppressive activity correlate with circulating 25(OH)D_3_ levels. This has been observed in patients with pancreatitis [6], multiple sclerosis [15], and asthma [16, 17] or those chronically infected with the hepatitis C virus [18]. Supplementation with vitamin D_3_ or an analogue increased T_Reg_ cell numbers in healthy individuals (140,000 IU oral vitamin D_3_/month) [19] or patients with undifferentiated connective tissue disease (0.5 *μ*g oral alfacalcidol/day) [20]. Other studies report a positive correlation between serum 1,25(OH)_2_D_3_ levels (but not 25(OH)D_3_ levels) and circulating T_Reg_ cell numbers in patients with multiple sclerosis [21]. A negative correlation between 25(OH)D_3_ and T_Reg_ cell numbers has been reported in cord blood [22]. Most studies support a positive relationship between circulating 25(OH)D_3_ levels and T_Reg_ cell activity; however, this has not always been associated with improved disease-related outcomes [15, 19].

Another intriguing aspect of vitamin D biology includes its ability to modulate the tropism of cells for certain tissues. Tropism for skin has been suggested in some studies, where 1,25(OH)_2_D_3_ or an analogue (nM range) increased the expression of the skin-tropic chemokine receptor CCR10 on cultured T cells [14, 23]. The 1,25(OH)_2_D_3_ analogue TX527 significantly upregulated other skin-homing molecules like CCR4 (but not CLA) on T cells, as well as inflammation-homing molecules (e.g., CCR5, CXCR3, and CXCR6) but downregulated expression of lymph node-homing molecules (CD62L, CCR7, and CXCR4) [23]. Serum 25(OH)D_3_ levels are associated with increased expression of the skin-tropic chemokine receptors CCR4 and CLA on circulating T_Reg_ cells from healthy male volunteers [24]. Other studies in HIV-infected participants suggested that serum 25(OH)D_3_ was negatively associated with CCR4 expression on circulating T_Reg_ cells. Vitamin D_3_ supplementation (25,000 IU/week) of these participants increased CCR4 and CCR10 expression on blood T_Reg_ cells [25]. Collectively, these studies suggest that 1,25(OH)_2_D_3_ promotes homing of T_Reg_ cells towards skin or sites of inflammation.

The results of a recent meta-analysis of clinical trials suggest that dietary vitamin D_3_ supplementation may reduce symptoms of atopic dermatitis [26], an inflammatory skin disease. In this study, we investigated how the tissue distribution and suppressive function of T_Reg_ cells are regulated by dietary vitamin D_3_. We used a murine model of dietary-induced vitamin D_3_ restriction to induce vitamin D_3_ deficiency and compared the effects of dietary vitamin D_3_ on T_Reg_ cell function and numbers in various tissues and skin inflammation induced by a hapten.

## 2. Materials and Methods

### 2.1. Mice and Diet

All experiments were performed according to the ethical guidelines of the National Health and Medical Research Council of Australia and with approval from the Telethon Kids Institute Animal Ethics Committee (AEC#229). BALB/c mice were purchased from the Animal Resources Centre, Western Australia. Mice transgenic for the OVA_323–339_-specific (ISQAVHAAHAEINEAGR) T cell receptor (DO11.10) on a BALB/c background were originally purchased from the Jackson Laboratory and bred in-house. Expression of OVA_323–339_-specific T cell receptor on T cells from DO11.10 mice was confirmed as previously described [9]. Female 3-week-old BALB/c or DO11.10 transgenic mice were placed on semipure diets, which were supplemented with vitamin D_3_ (2280 IU vitamin D_3_/kg with 1% calcium, SF05-34, Specialty Feeds, Perth, Western Australia) or were not supplemented with vitamin D_3_ (0 IU vitamin D_3_/kg with 2% calcium, SF05-033, Specialty Feeds) as previously described [27, 28]. At 8 weeks of age, female mice were mated with adult male mice maintained on standard mouse chow (Specialty Feeds, containing 2000 IU vitamin D_3_/kg). Female or male offspring were maintained on the same vitamin D_3_-replete or vitamin D_3_-deficient diets (as their mothers) for the rest of the experiment. All results shown are for female offspring, unless otherwise stated.

### 2.2. Measurement of Serum 25-Hydroxyvitamin D_3_ (25(OH)D_3_)

Serum 25(OH)D_3_ levels were measured in BALB/c mice using IDS EIA ELISA kits (Immunodiagnostic Systems Ltd., Fountain Hills, AZ) as described by the manufacturer (limit of detection was 7 nmol·L^−1^). We have previously shown that results from this assay correlate highly (*r* = 0.99) [29] with a liquid chromatography-tandem mass spectrometry method, which has been certified to a reference measurement procedure developed by the National Institute of Standards and Technology and Ghent University [30, 31].

### 2.3. Biphasic Ear Swelling Assay

A biphasic ear swelling response [32, 33] was induced by painting both sides of each ear pinnae with 10 *μ*L of 0.05–0.2% 2,4-dinitrofluorobenzene (DNFB, Sigma, St Louis, MO) in acetone using a micrometer to measure ear thickness in a blinded fashion at the indicated times. Results are shown as the change in ear thickness, with baseline measures subtracted from those measured at each time point.

### 2.4. Identification of T_Reg_ Cells by Flow Cytometry

Single cell suspensions were generated from minced ear skin or whole lung digested for 90 min with collagenase IV (3 mg/mL, Worthington) at 37°C with frequent vortexing. To isolate peripheral blood mononuclear cells (PBMC), heparinized blood was diluted 1 : 2 in 0.9% saline (Baxter, Old Toongabbie, NSW, Australia) and layered over 1 mL Lymphoprep (Axis-Shield, Oslo, Norway) for every 2 mL of diluted blood. Samples were then centrifuged at 800 ×g for 20 min at room temperature with PBMCs collected from the resulting interface. Skin-draining lymph nodes (SDLN; brachial, inguinal, and axillary), airway-draining lymph node cells (ADLN; posterior mediastinal, tracheobrachial, and parathymic), mesenteric lymph nodes (MLN), or spleens were removed from mice and physically disaggregated to generate single cell suspensions as previously described [9]. Staining of surface (CD3, CD4, CD25, CCR4, CCR10, neuropilin, MHC class II, and CD11c) and intracellular (Foxp3) antigens was performed as previously described [9]. At least 10,000 cells of interest were collected using the FACS LSRII (BD Biosciences) flow cytometer. Data were analyzed using FlowJo software (v9.5.2, TreeStar Inc., Ashland, OR, USA).

### 2.5. Assessing the Suppressive Capacity of T_Reg_ Cells

We isolated T_Reg_ cells from vitamin D_3_-replete or vitamin D_3_-deficient DO11.10 mice to test the capacity of dietary vitamin D_3_ to modify the suppressive activity of cells located in a number of different immune sites. As the majority of T_Reg_ cells express the OVA_323–339_ receptor [9], they will suppress the IL-2-secreting capacity of cocultured OVA_323–339_ receptor-specific CD4+ T cells in the presence of antigen-presenting cells and OVA_323–339_ peptide, as we have demonstrated previously [9]. CD4+CD25+ cells (≥95% pure, as determined by flow cytometry) were isolated from specified tissues of DO11.10 mice using a CD4+CD25+ regulatory T cell isolation kit (Miltenyi Biotec) or by cell sorting as previously described [9, 34]. Greater than 90% of the purified CD4+CD25+ cells expressed Foxp3 (confirmed by flow cytometry). Peripheral lymph node cells (including SDLN, ADLN, MLN, auricular-draining lymph nodes, and para-aortic lymph nodes) of naïve DO11.10 mice were used as responder cells. These were resuspended in RPMI with 10% FCS and 2 *μ*M 2-ME and aliquot into round-bottomed 96-well plates at 10^5^ cells/0.1 mL/well. CD4+CD25+ cells were added to responder cells at ratios of 1 : 8, 1 : 16, or 1 : 32. OVA_323–339_ peptide was added at a final concentration of 1 *μ*g/mL. After incubation for 92 h at 37°C in 5% CO_2_, supernatants were harvested and the concentration of interleukin-2 (IL-2) was determined by ELISA as previously described [9].

### 2.6. Assessing the Ability of Dendritic Cells to Induce T_Reg_ Cells

A single cell suspension of ADLN cells was prepared by physically disaggregating lymph nodes and digesting samples with collagenase IV (1 mg/mL, Worthington) and DNase I (0.1 mg/mL, Sigma) for 30 min at 37°C. Conventional DCs were enriched from the ADLN cells by removal of CD3+, Thy1.1+ CD19+, GR-1+, and TER-119+ cells using magnetic bead separation as previously described [35]. The remaining cells were then labelled with antibodies specific for CD11c and MHC class II [9] and MHC class II^hi^CD11c^med^ cells sorted by FACS using the FACSAria (BD Biosciences). MHC class II^hi^CD11c^med^ cells were incubated with peripheral lymph nodes from naïve DO11.10 (vitamin D-replete) mice (see [9]) at a ratio of 1 : 40 with 1 *μ*g/mL OVA_323–339_ peptide. DCs were not added to some cultures as controls. Cells were incubated for 62 h at 37°C and 5% CO_2_, and then CD3+CD4+CD25+Foxp3± cells were identified by flow cytometry using the FACS LSRII, where at least 5,000 Foxp3+ cells were collected. Data were analyzed using FlowJo software.

### 2.7. Statistical Analyses

Data were compared using an unpaired two-way Student's *t*-test using Prism 5 statistical analysis program for Mac OS X. Differences were considered significant with a *p* value < 0.05. Data are shown throughout as mean ± SEM.

## 3. Results and Discussion

### 3.1. Vitamin D Deficiency Promoted Allergic Dermatitis Responses Measured during a Biphasic Ear Swelling Response

**Figure 1:**
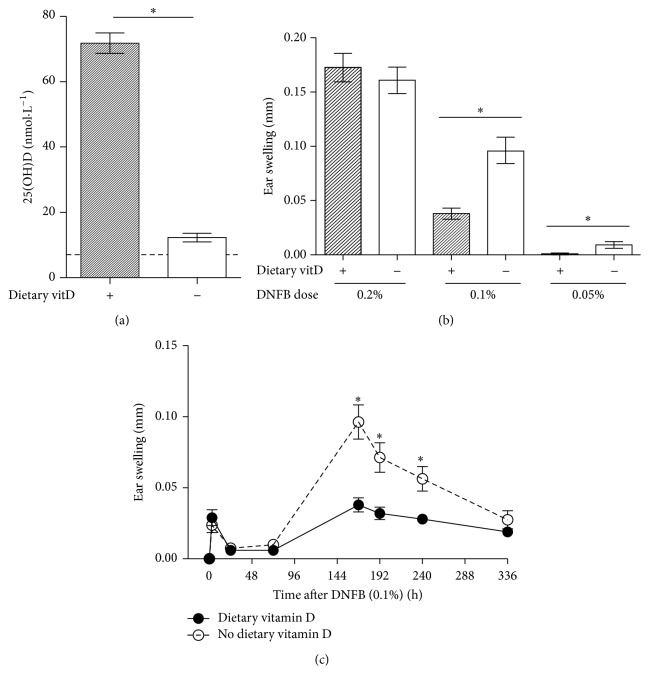
Dietary vitamin D suppressed the biphasic ear swelling response induced by the hapten DNFB. Female offspring born to vitamin D_3_- (vitD-) replete (+) or vitamin D_3_-deficient (−) BALB/c mothers were maintained on vitamin D_3_-replete or vitamin D_3_-deficient diets (resp.). (a) Serum 25(OH)D_3_ levels of female offspring at 8 weeks of age (mean ± SEM for ≥5 mice per group). The broken line indicates the level of detection for 25(OH)D_3_ (7 pg/mL). In (b), the ear pinnae of mice were sensitized with 0.2, 0.1, or 0.05% DNFB and the ear swelling response was measured at the second peak of the biphasic response (144 h). In (c), the ear pinnae of mice were sensitized with 0.1% DNFB and the resulting biphasic ear swelling response was measured over 305 h. Data are shown as mean ± SEM in (b) and (c) for 8–12 ear pinnae of 4–6 mice per treatment (^*∗*^ *p* < 0.05). In (b), data are from a single experiment and in (c) they are representative of 2 experiments.

We investigated the effects of dietary vitamin D on allergic dermatitis responses mimicked by inducing a biphasic ear swelling response. We tested adult female offspring of vitamin D_3_-replete or vitamin D_3_-deficient BALB/c dams, which were maintained on the same diet as their mothers. Serum levels of 25(OH)D_3_ were <20 nmol·L^−1^ for offspring fed the vitamin D_3_-deficient diet and >50 nmol·L^−1^ for offspring fed the vitamin D_3_-replete diet (Figure 1(a)). These diets did not significantly alter serum calcium [27, 28]. The contact sensitizer DNFB was then used to initiate a biphasic ear swelling response [32, 33]. The ears of vitamin D_3_-deficient or vitamin D_3_-replete mice were sensitized with 0.05–0.2% DNFB (in acetone), and ear swelling was recorded over a 3-week period. The ability of dietary vitamin D_3_ to suppress ear swelling responses depended on the sensitizing dose of DNFB, where responses to ≤0.1% DNFB were suppressed at 144 h after sensitization, corresponding to the second peak of ear swelling (Figure 1(b)). As expected, the ear swelling response was biphasic, with an initial peak at 3 h and later peak at 168 h after DNFB treatment (Figure 1(c)). Previous studies have shown that this first peak represents an early innate influx of neutrophils and inflammatory cells into ear skin, which may depend on histamine release by mast cells [32], while the second peak is an antigen-specific (DNFB) effector response driven by CD8+ T cells expressing interferon-*γ* [33]. Dietary vitamin D_3_ significantly suppressed (by 61%) this second “efferent” ear swelling response, which peaked at 168 h after DNFB application in vitamin D_3_-sufficient mice as compared to responses observed in deficient mice (Figure 1(c)).

### 3.2. Increased Percentages of T_Reg_ Cells Were Observed in the Skin-Draining Lymph Nodes of Vitamin D_3_-Replete Mice

**Figure 2:**
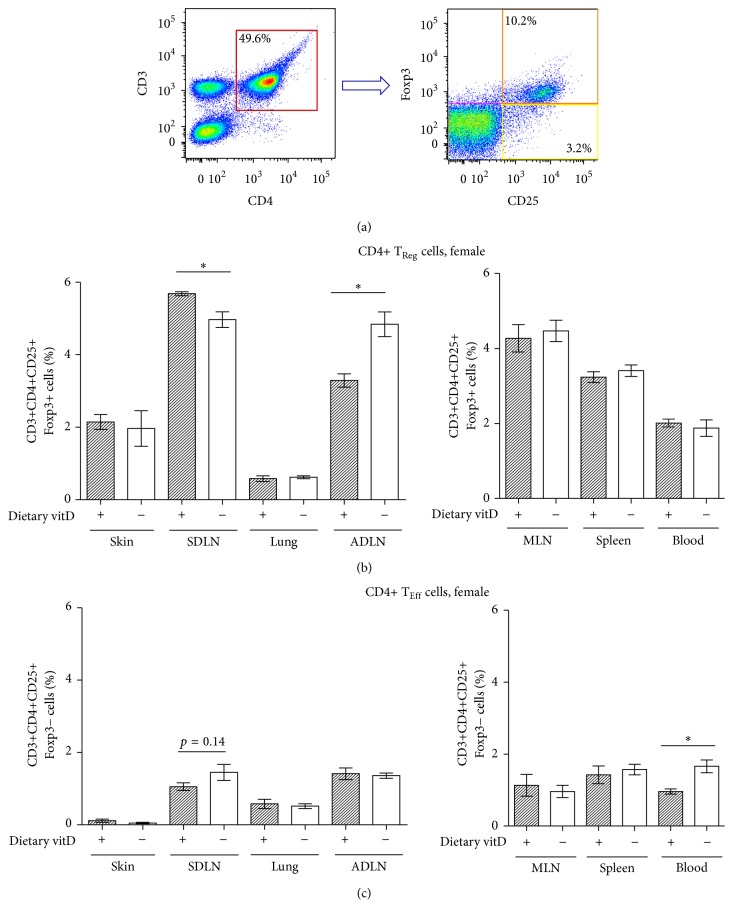
Dietary vitamin D_3_ increased the percentage of Foxp3+ T_Reg_ cells in the SDLN. Female offspring born to vitamin D_3_- (vitD-) replete (+) or vitamin D_3_-deficient (−) BALB/c mothers were maintained on the vitamin D_3_-replete or vitamin D_3_-deficient diets (resp.). (a) The FACS gating strategy for determining the percentage of CD3+CD4+CD25+Foxp3+ (T_Reg_ cells, orange) or CD3+CD4+CD25+Foxp3− (T_Eff_ cells, yellow) cells in skin, SDLN, lung, ADLN, MLN, spleen, and blood. Representative plots from a MLN sample are shown. For all tissues, a gate was used to exclude red blood cells using forward and side scattering properties of cells prior to selection of the various T cell populations. (b) and (c) The percentage of T_Reg_ cells and T_Eff_ cells (resp.) in various tissues. Data are shown as mean ± SEM for 6 mice/treatment with results combined from two experiments; ^*∗*^ *p* < 0.05.

We have previously published that topically applied 1,25(OH)_2_D_3_ increased the capacity of T_Reg_ cells to suppress contact hypersensitivity responses initiated by DNFB [9]. To examine the effects of dietary vitamin D_3_ on T_Reg_ cells, their percentages in various tissues were measured in naïve female mice prior to sensitization with DNFB. The percentages of CD3+CD4+CD25+Foxp3+ T_Reg_ cells in the skin, SDLN, lung, ADLN, MLN, spleen, and blood were determined by flow cytometry (Figure 2(a), a representative plot for a MLN sample is shown). CD4+ T_Reg_ cell percentages were increased in the SDLN (from 5.0 ± 0.2 (vitD−) to 5.7 ± 0.1 (vitD+); 14% increase; *n* = 6/treatment) and reduced in the ADLN (from 4.8 ± 0.3 (vitD−) to 3.3 ± 0.2 (vitD+); 31% reduction; *n* = 6/treatment) of vitamin D_3_-replete mice in comparison to vitamin D_3_-deficient mice, but there was no difference in the percentages of these cells in the skin, lungs, MLN, spleen, or blood (Figure 2(b)). There was also a trend (*p* = 0.08, Student's *t*-test) for increased Foxp3 expression (by 16%) on CD3+CD4+CD25+Foxp3+ cells from the SDLN of vitamin D_3_-replete mice, when geometric mean fluorescence intensity was compared (1053 ± 43 (vitD+); 910 ± 63 (vitD−); *n* = 6/treatment, data from cells collected in Figure 2(b)). There was no difference in the expression of Foxp3 on CD3+CD4+CD25+Foxp3+ cells from the other tissues (data not shown). With the exception of blood, CD3+CD4+CD25+Foxp3− T “effector” cell (T_Eff_) (Figure 2(c)) percentages were unaffected by vitamin D_3_ deficiency. In male mice, CD3+CD4+CD25+Foxp3+ T_Reg_ cell percentages were affected in a similar way by dietary vitamin D_3_ as those observed in female mice and were increased in the SDLN (by 21%) (from 4.1 ± 0.3 (vitD−) to 5.0 ± 0.2 (vitD+); *n* = 6/treatment) and reduced in ADLN (by 23%) (from 4.3 ± 0.3 (vitD−) to 3.3 ± 0.1 (vitD+); *n* = 6/treatment). There was also an increase (42%) in the percentage of CD3+CD4+CD25+Foxp3−  T_Eff_ cells in the SDLN of male mice fed a vitamin D_3_-containing diet (from to 0.31 ± 0.04 (vitD−) to 0.44 ± 0.04 (vitD+); 42% increase; *n* = 6/treatment).

**Figure 3:**
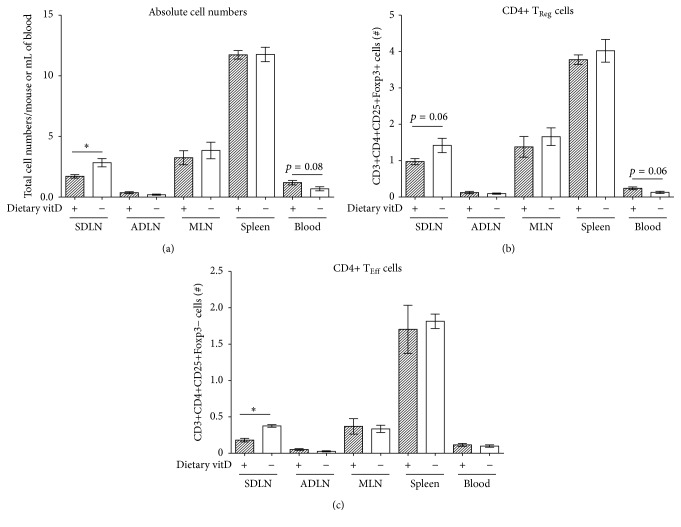
Dietary vitamin D_3_ reduced the absolute number of cells in the SDLN. Female offspring born to vitamin D_3_- (vitD-) replete (+) or vitamin D_3_-deficient (−) BALB/c mothers were maintained on the vitamin D_3_-replete or vitamin D_3_-deficient diets (resp.). In (a), the number of cells (×10^7^)/mouse or mL of blood isolated from the SDLN, ADLN, MLN, spleen, or blood is shown. The numbers of CD3+CD4+CD25+Foxp3+ (T_Reg_ cells) or CD3+CD4+CD25+Foxp3− (T_Eff_ cells) cells in these tissues were calculated using the percentages depicted in Figure 2. In (b) and (c), the number of CD4+ T_Reg_ and T_Eff_ cells (×10^6^)/mouse or mL of blood is shown. Data are shown as mean ± SEM for 6 mice/treatment with results combined from two experiments; ^*∗*^ *p* < 0.05.

The number of cells isolated from the SDLN was altered by vitamin D_3_ supplementation of female mice. Significantly fewer SDLN cells (2.8 ± 0.4 × 10^7^/mouse (vitD−); 1.7 ± 0.1 × 10^7^/mouse (vitD+); 39% reduction; *n* = 6/treatment) were isolated from vitamin D_3_-supplemented mice (Figure 3(a)). These effects were in the opposite direction to those of dietary vitamin D_3_ on T_Reg_ cell percentages in the SDLN. There was no difference in the numbers of cells isolated from other tissues (Figure 3(a); data not shown for skin and lung). There was a trend for fewer CD4+ T_Reg_ cell numbers in the SDLN (1.5 ± 0.2 × 10^6^/mouse (vitD−); 0.9 ± 0.1 × 10^6^/mouse (vitD+)); 40% reduction; *n* = 6/treatment) of vitamin D_3_-supplemented mice in comparison to vitamin D_3_-deficient mice (Figure 3(b)). Similarly, numbers of CD3+CD4+CD25+ Foxp3−  T_Eff_ cells were significantly reduced in the SDLN of mice fed a vitamin D_3_-supplemented diet (3.8 ± 0.2 × 10^5^/mouse (vitD−); 1.8 ± 0.2 × 10^5^/mouse (vitD+); 53% reduction; *n* = 6/treatment) (Figure 3(c)). There was no effect of dietary vitamin D_3_ on the total cell numbers or numbers of T_Reg_ or T_Eff_ cells identified in the SDLN, ADLN, or blood of male mice (data not shown). These data suggest that while the proportions of CD4+ T_Reg_ cells increased in the SDLN with dietary vitamin D_3_, this significant increase did not persist when cell numbers were considered, as significantly fewer SDLN cells were isolated from mice fed a diet containing vitamin D_3_.

### 3.3. The Suppressive Activity of T_Reg_ Cells Was Enhanced by Dietary Vitamin D_3_ in Most Immune Tissues but Not the Airway-Draining Lymph Nodes

**Figure 4:**
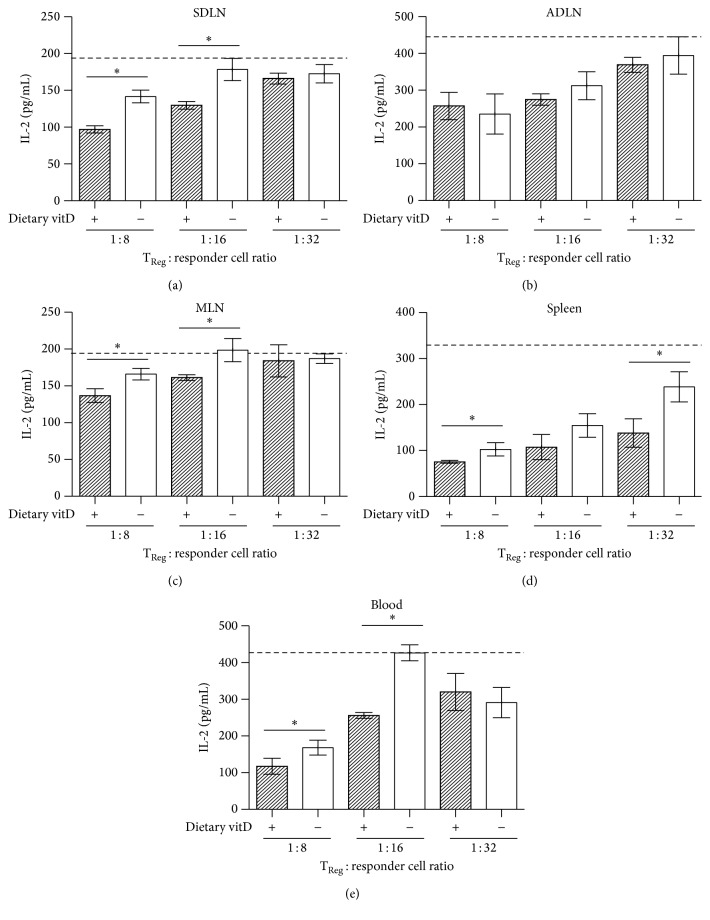
Dietary vitamin D enhanced the activity of Foxp3+ T_Reg_ cells at multiple immune sites. Female offspring born to vitamin D_3_- (vitD-) replete (+) or vitamin D_3_-deficient (−) DO11.10 mothers were maintained on vitamin D-replete or vitamin D-deficient diets (resp.) until 8 weeks of age. CD4+CD25+(Foxp3+) cells were purified from (a) SDLN, (b) ADLN, (c) MLN, (d) spleens, or (e) blood of mice and cocultured with lymph node responder cells from DO11.10 mice at ratios of 1 : 8, 1 : 16, or 1 : 32 and OVA peptide. IL-2 levels in the coculture supernatants were measured after 96 h. The broken lines indicate the levels of IL-2 measured in supernatant of responder cells cultured with OVA peptide alone. Data are shown as mean ± SEM for 6 wells/treatment, representative of two experiments; ^*∗*^ *p* < 0.05.

An* in vitro* assay was used to test if dietary vitamin D_3_ altered the suppressive function of T_Reg_ cells in comparison to those from vitamin D_3_-deficient mice. Purified CD4+CD25+(Foxp3+) cells were cocultured with responder lymph node cells from DO11.10 mice and OVA_323–339_ peptide for 92 h. Responder CD4+ T cells expressing the OVA_323–339_ TCR proliferate and produce cytokines like IL-2 in response to presentation of the OVA_323–339_ peptide by antigen-presenting cells. We assessed IL-2 levels as a measure of the proliferative capacity of responder cells, where cocultured T_Reg_ cells significantly suppressed supernatant levels of IL-2 in a dose-dependent manner (Figure 4). T_Reg_ cells produce very low levels of IL-2 when stimulated* in vitro*. These levels are up to 10 times less than CD4+ T_Eff_ responder cells [12]. T_Reg_ cells therefore do not significantly contribute towards the pool of IL-2 in coculture assays. CD4+CD25+(Foxp3+) cells from the SDLN (Figure 4(a)), MLN (Figure 4(c)), spleen (Figure 4(d)), and blood (Figure 4(e)) of vitamin D_3_-replete mice had increased capacity to suppress IL-2 production by cocultured responder cells. There was no significant difference in suppressive function observed for CD4+CD25+(Foxp3+) cells from the ADLN (Figure 4(b)) of vitamin D_3_-replete or vitamin D_3_-deficient mice. The results reported in Figure 4 were for suppressive activities of CD4+CD25+(Foxp3+) cells from female mice; however, similar results were obtained for cells from male mice (data not shown). We were not technically able to assess the suppressive activity of T_Reg_ cells in the skin or lungs as their numbers were too infrequent for efficient isolation. These data suggest that dietary vitamin D_3_ is required for the optimal activity of T_Reg_ cells at various immune sites throughout the body, with the exception of the ADLN.

### 3.4. Vitamin D_3_ Did Not Induce T_Reg_ Cells in the Periphery

**Figure 5:**
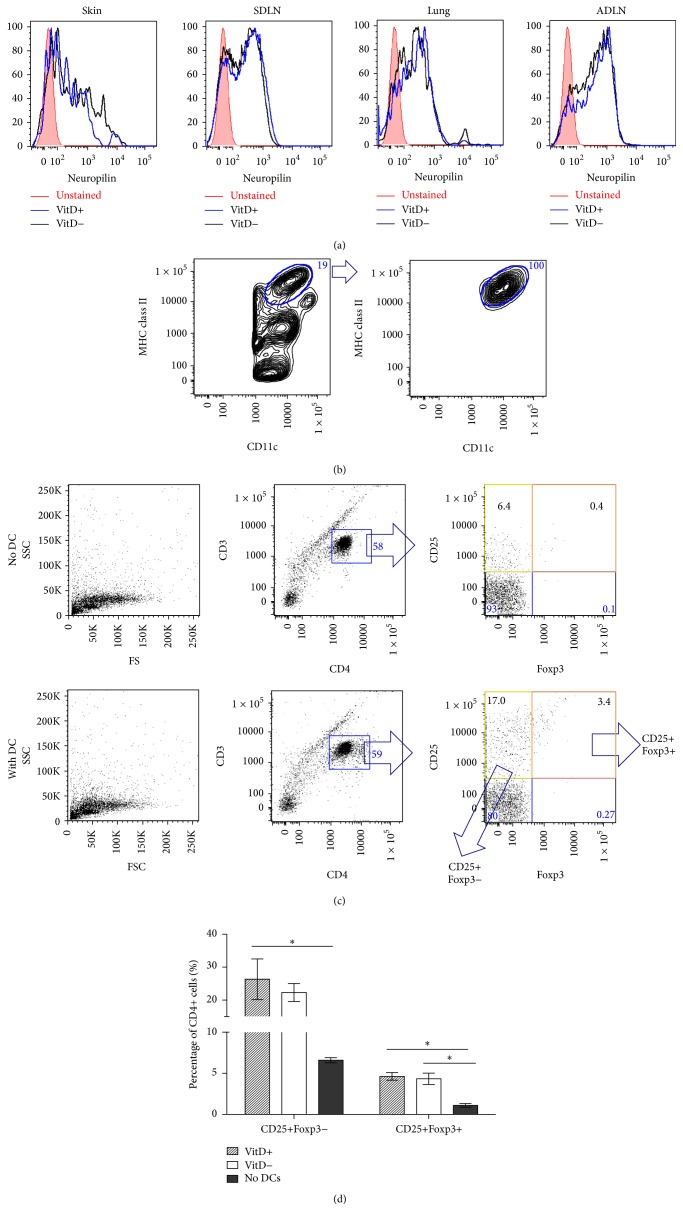
Dietary vitamin D_3_ did not modify neuropilin expression on Foxp3+ T_Reg_ cells from the skin, SDLN, lung, or ADLN or alter the capacity of DCs from the ADLN to induce new Foxp3+ T_Reg_ cells. Female offspring born to vitamin D_3_-replete or vitamin D_3_-deficient BALB/c mothers were maintained on the vitamin D_3_-replete (vitD+) or vitamin D_3_-deficient (vitD−) diets (resp.) until 8 weeks of age. In (a), the expression of neuropilin in the skin, SDLN, lungs, and ADLN is shown, with cells from vitamin D_3_-replete and vitamin D_3_-deficient mice shown in blue and black, respectively (unstained = red shaded). Data are representative of two experiments. In (b), MHC class II^hi^CD11c^med^ cells from the ADLN were sorted with the prepurity (left) and postpurity (right) shown, as determined by flow cytometry. These cells were cocultured with lymph node cells from OVA-TCR transgenic mice (DO11.10 mice) at a ratio of 0 : 1 (no DC) or 1 : 40 (with DC) for 62 h with OVA peptide. (c) The gating strategy for determining the percentage of CD25+Foxp3− and CD25+Foxp3+ cells after 62 h of coculture. In (d), the percentages of CD25+Foxp3− and CD25+Foxp3+ cells of CD4+CD3+ cells are shown as mean ± SEM for 3 wells/treatment. ^*∗*^ *p* < 0.05.

Surface expression of neuropilin can be used to identify naturally occurring T_Reg_ cells [36]. We examined the expression of neuropilin on T_Reg_ cells from the skin, SDLN, lungs, or ADLN of vitamin D_3_-replete or vitamin D_3_-deficient mice and observed no difference in the expression of this molecule (Figure 5(a)). These results suggest that dietary vitamin D_3_ did not favour the induction of new T_Reg_ cells in the SDLN. The observed reduction in the percentage of T_Reg_ cells in the ADLN of mice fed a vitamin D_3_-containing diet was a surprising result. A lack of difference in neuropilin expression on T_Reg_ cell from the ADLN of vitamin D_3_-replete and vitamin D_3_-deficient mice suggested that dietary vitamin D_3_ did not prevent the induction of new T_Reg_ cells (Figure 5(a)). However, to confirm this observation, we then tested whether there was a functional difference between DCs from vitamin D_3_-deficient and vitamin D_3_-replete mice, as DCs are required for the induction of new T_Reg_ cells in the periphery [37]. Conventional MHC class II^hi^CD11c^med^ DCs were sorted (Figure 5(b)) from the ADLN of vitamin D_3_-replete and vitamin D_3_-deficient mice and cocultured with lymph node cells from naïve DO11.10 mice and OVA peptide. The percentage of CD4+CD25+Foxp3± cells was determined after 62 h of coculture (Figure 5(c)). CD4+CD25+Foxp3−  T_Eff_ cell and CD4+CD25+Foxp3+ T_Reg_ cell percentages were increased (>2-fold) by the presence of DCs in the cocultures (Figures 5(c) and 5(d)). However, there was no effect of dietary vitamin D_3_ on the ability of ADLN DCs to regulate T_Reg_ cell percentages (Figure 5(d)). These results suggest that dietary vitamin D_3_ did not impair the induction of new T_Reg_ cells in the ADLN.

### 3.5. Dietary Vitamin D_3_ Did Not Affect the Expression of CCR4 or CCR10 on T_Reg_ Cells in the SDLN or ADLN

**Figure 6:**
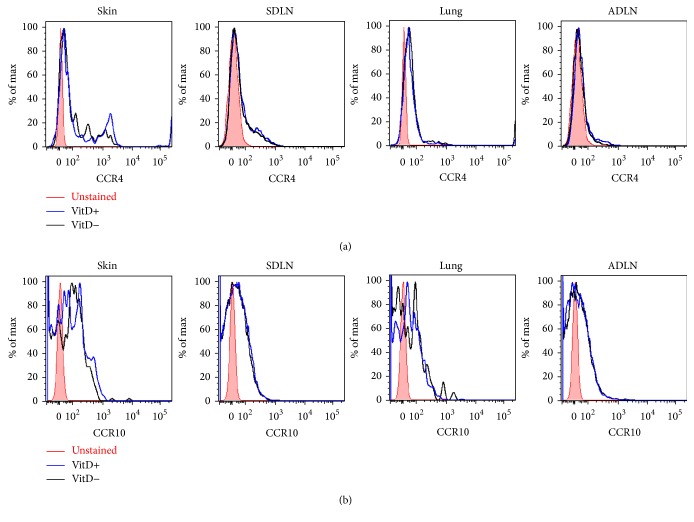
Dietary vitamin D_3_ did not modify CCR4 or CCR10 expression on Foxp3+ T_Reg_ cells from the skin, SDLN, lung, or ADLN. Female offspring born to vitamin D_3_-replete or vitamin D_3_-deficient BALB/c mothers were maintained on the vitamin D_3_-replete (VitD+) or vitamin D_3_-deficient diets (VitD−) (resp.) until 8 weeks of age. The expression of (a) CCR4 and (b) CCR10 was measured on CD3+CD4+CD25+Foxp3+ cells in the skin, SDLN, lungs, and ADLN, with cells from vitamin D_3_-replete and vitamin D_3_-deficient mice shown in blue and black, respectively (unstained = red shaded). Data are representative of two experiments.

Dietary vitamin D_3_ could induce the migration of T_Reg_ cells to augment the percentages of these cells in the SDLN, facilitated by the expression of chemokine receptors. There was no difference in the expression of the skin-homing receptors CCR4 or CCR10 on T_Reg_ cells in the skin, SDLN, lungs, or ADLN of vitamin D-replete or vitamin D-deficient mice (Figures 6(a) and 6(b)). Significant levels of CCR4 (Figure 6(a)) were detected on T_Reg_ cells in skin and SDLN with less expression on cells from the lung and ADLN, while similar levels of CCR10 (Figure 6(b)) were identified on T_Reg_ cells from these tissues. While dietary vitamin D_3_ promoted T_Reg_ cell accumulation in the SDLN, there was no difference in the expression of CCR4 and CCR10 skin-homing receptors once cells entered the SDLN.

### 3.6. Dietary Vitamin D_3_ May Promote Dermal Tolerance to Reduce Skin Inflammation

Our findings of the immunosuppressive effects of dietary vitamin D_3_ in controlling DNFB-induced skin inflammation reiterate emerging data from clinical trials, which suggest that vitamin D_3_ supplementation reduces symptoms of allergic dermatitis [26]. Similar suppressive effects of dietary vitamin D_3_ have been observed in other animal models that used haptens to induce skin inflammation [38]. In other studies, hypocalcaemia induced by vitamin D_3_ deficiency may have impaired hapten-induced ear swelling responses [39]. The vitamin D_3_-containing diets used in our studies and those of others [38] were enriched with calcium to prevent hypocalcemia [27, 28]. While clinical trials suggest that maintaining optimal serum levels of 25(OH)D through dietary vitamin D_3_ supplementation (or safe sun exposure) reduces symptoms of atopic dermatitis [26], we are still uncertain of the optimal levels of 25(OH)D needed to limit atopic dermatitis. It is also important to note that some studies have observed no significant effect of vitamin D_3_ supplementation [40], with suggestions that genetic or other population-based factors (e.g., initial circulating 25(OH)D levels) or the supplementation regimen (e.g., dose, schedule of treatment, and type of vitamin D) may have limited the efficacy of the vitamin D treatment.

### 3.7. Dietary Vitamin D_3_ Increased the Percentages and Activity of T_Reg_ Cells in the SDLN

We observed a greater suppressive activity of CD4+CD25+(Foxp3+) cells isolated from the SDLN, MLN, spleen, and blood of mice fed the vitamin D-containing diet, suggesting a systemic effect of dietary vitamin D on T_Reg_ cell activity. It is important to note that we examined T_Reg_ cell numbers and function prior to sensitization with DNFB, and so these findings are independent of skin inflammation induced by the irritant. These observations were accompanied by a lack of effect of dietary vitamin D on the suppressive activity of cells from the ADLN. This curious observation is difficult to explain but may represent a site from which T_Reg_ cells actively migrate (to the SDLN). We did not observe increased expression of skin-tropic chemokine receptors CCR4 or CCR10 on T_Reg_ cells from any of the tested sites (skin, SDLN, lung, and ADLN) of vitamin D_3_-fed mice, but it may be that these molecules are upregulated during transition (in blood) between locations, which could be a focus of future studies. Crosstalk between the immune reactions initiated in the skin and airways is illustrated by the “atopic march” concept, where allergic responses in the skin affect immunity in the airways. Indeed, hapten-induced skin inflammation can worsen signs of allergic airway disease in mice [41]. We suggest that dietary vitamin D_3_ may prevent the “atopic march” by promoting T_Reg_ cell accumulation and activity in the SDLNs. The lack of difference in neuropilin levels suggests that vitamin D_3_ may increase SDLN T_Reg_ cell accumulation through migration.

### 3.8. Inconsistencies between These Observations and Other Published Data

Urry et al. (2012) observed a positive correlation between circulating 25(OH)D and the percentages of T_Reg_ cells in the airways (lavage fluid) of children with severe asthma [42]. We did not see any difference in the percentages of T_Reg_ cells in the lungs of mice fed a vitamin D_3_-replete or vitamin D_3_-deficient diet and did not assess the percentages of these cells in the trachea or bronchoalveolar lavage fluid. Furthermore, reduced percentages of T_Reg_ cells were observed in the ADLN of mice fed a vitamin D_3_-replete diet. In addition, it is possible that, upon respiratory stimulation with allergen, T_Reg_ cell percentages could increase in the lungs of vitamin D_3_-replete mice. In other studies, Mann et al. (2015) found that more CD4+ cells stimulated with 1,25(OH)_2_D (100 nM)* in vitro *expressed neuropilin compared to control cells [43]. It is therefore possible that new T_Reg_ cells may be generated under conditions of highly concentrated 1,25(OH)_2_D_3_.

### 3.9. Modelling Skin Inflammation

We induced a biphasic cutaneous skin reaction using the contact sensitizer DNFB to examine the effects of dietary vitamin D_3_ on skin inflammation. A humanized mouse model would have been an interesting alternative means of comparing these treatments through xenotransplantation of human skin [44] or bioengineered human skin equivalents [45] onto immunodeficient mice. These models can induce some (but not all) clinical manifestations of atopic dermatitis, particularly acute lesions [45]. While such models would improve our understanding of the effects of dietary vitamin D_3_ in humanized settings, we were more interested in the capacity of dietary vitamin D_3_ to modulate T_Reg_ cell proportions and function in certain tissues (e.g., skin or lungs) prior to the onset of inflammation.

## 4. Conclusion

Our studies suggest that dietary vitamin D_3_ increased the percentages and suppressive function of T_Reg_ cells in the SDLN and that these cells are poised to suppress dermal inflammation. These studies support the notion that maintaining adequate serum 25(OH)D through dietary vitamin D_3_ supplementation or safe sun exposure is important to reduce the severity of allergic dermatitis and other inflammatory skin conditions.

## Abbreviations

1,25(OH)_2_D_3_: 1,25-Dihydroxyvitamin D_3_
25(OH)D_3_: 25-Hydroxyvitamin D_3_
ADLN: Airway-draining lymph nodes
DNFB: 2,4-Dinitrofluorobenzene
T_Eff_ cells: Effector T cells
MLN: Mesenteric lymph nodes
T_Reg_ cells: Regulatory T cells
SDLN: Skin-draining lymph nodes
VDBP: Vitamin D-binding protein.
